# Obesity is associated with adverse outcomes in primary immune thrombocytopenia - a retrospective single-center study

**DOI:** 10.1007/s00277-024-05836-3

**Published:** 2024-06-12

**Authors:** Zhengrui Xiao, Zhiqiang He, Hieu Liem Le Nguyen, Rahul Kumar Thakur, M. Bakri Hammami, Hiba Narvel, Charan Thej Reddy Vegivinti, Noelle Townsend, Henny Billett, Irina Murakhovskaya

**Affiliations:** 1 Division of Hematology, Department of Hematology-Oncology, Montefiore Medical Center, Albert Einstein College of Medicine, Bronx, NY 10467 USA; 2 School of Public Health, Nanjing Medical University, Nanjing, 211166 China; 3 Department of Internal Medicine, Jacobi Medical Center, Albert Einstein College of Medicine, Bronx, NY 10461 USA; 4 Department of Laboratory Medicine and Pathology, Mayo Clinic, Rochester, MN 55905 USA

**Keywords:** ITP, Thrombocytopenia, Obesity, Outcomes, Prognosis

## Abstract

The pathophysiology of immune thrombocytopenia (ITP) involves immune-mediated platelet destruction. The presence of adipose tissue in obese individuals creates an inflammatory environment that could potentially impact the clinical course and outcomes of ITP. However the relationship between obesity and ITP outcomes has not been well described. We evaluated ITP outcomes in 275 patients diagnosed with primary ITP from 2012 to 2022. Patients were categorized into four groups based on their body mass index (BMI) at diagnosis. Female gender was associated with a lower platelet count at the time of diagnosis at any BMI. Patients with high BMI had lower platelet counts at diagnosis and at platelet nadir (*p* < 0.001), an increased likelihood of requiring therapy (*p* < 0.001) and requiring multiple lines of therapy (*p* = 0.032). Non-obese patients who required corticosteroid treatment experienced a longer remission duration compared to obese patients (*p* = 0.009) and were less likely to be steroid-dependent (*p* = 0.048). Our findings suggest that obesity may be a significant risk factor for developing ITP and for ITP prognosis. Future studies are needed to evaluate the role of weight loss intervention in improving ITP outcomes.

## Introduction

Immune thrombocytopenic purpura (ITP) is a rare hematologic disorder in adults, with an incidence between 1.6/100,000 to 2.25/100,000 [1, 2]. It is an acquired disease that presents as a decrease in the platelet count and rarely bleeding, depending on the degree of thrombocytopenia [3]. While formerly known as idiopathic thrombocytopenic purpura, the immune-mediated mechanisms are now well understood, involving antiplatelet autoantibody production, T-helper cell 1 activation, cytokine over-secretion, direct cytotoxicity effect via T-lymphocytes and natural killer cells and complement-mediated platelet destruction [4, 5]. This immune response is favored by deficiency of regulatory T cells [6]. In addition, there is inadequate bone marrow production of platelets due to antibody-mediated inhibition of megakaryocyte production [7] as well as insufficient production of thrombopoietin [8, 9].

The adipokines produced by adipose tissue, including leptin, adiponectin, resistin, and visfatin, contribute to the pathogenesis of various immune-mediated conditions such as rheumatoid arthritis, systemic lupus erythematosus, inflammatory bowel disease and multiple sclerosis [10]. However, studies evaluating a potential association between obesity and ITP are scarce [9, 11].

Obesity has become a growing public health problem in the United States and can cause serious health and financial consequences. Patients diagnosed with ITP also experience substantial economic burdens which affect their quality of life [12, 13]. If obesity is a significant risk factor for ITP, there may be a place for weight reduction therapies to mitigate this immune disease. With high rates of obesity (32.9%) and poverty in the Bronx (26%) [14], we proposed to investigate the possible relationship between obesity and ITP outcomes.

## Materials and methods

### Study design and patient population

A retrospective cohort study was conducted at Montefiore Medical Center, a tertiary academic inner city medical facility in the Bronx, New York. Adult patients over the age 18 diagnosed with ITP between April 2010 and April 2022 were included in the study. In order to minimize the effect of confounding factors, our study only focused on primary ITP. Patients were included if they had a platelet count of < 150 × 10^9^/L with no other recognized causative etiology. We excluded patients who met any of the following exclusion criteria: (1) thrombocytopenia in the setting of other conditions such as liver disease, bone marrow failure, myelodysplastic syndrome, acute myeloid leukemia, chemotherapy-induced thrombocytopenia, hypersplenism, (2) secondary ITP including viral etiology and recently coronavirus disease (COVID-19) related, autoimmune conditions (autoimmune diseases or positive rheumatologic serology without a definitive autoimmune disease diagnosis), pregnancy-associated thrombocytopenia, H pylori infection, lymphoproliferative disorders, (3) missing and/or insufficient data and patients who were underweight. Patient follow-up for a minimum of 1 month was required.

This study was approved by the institutional review board (IRB) of Albert Einstein College of Medicine.

### Data extraction

We used the keywords “thrombocytopenia”, “immune thrombocytopenic purpura” and “idiopathic thrombocytopenic purpura” to identify patients’ data from Electronic Medical Record (EMR) system. Patients’ baseline demographic data included self-identified gender and ethnicity, body mass index (BMI) at diagnosis. Laboratory data were collected at diagnosis and at nadir throughout the course as were outcome data [treatment requirement, response to initial therapy, duration of response (months), number of lines of treatment, time to achieve response (weeks), bleeding (major, minor), corticosteroid-dependence, refractoriness] were collected. Definitions of diagnosis and response, including partial response (PR), complete response (CR), corticosteroid-dependence and refractoriness were based on the ITP International Working Group [3]. The definition of major bleeding and clinically relevant non-major (CRNM) bleeding is based on the statement of the International Society on Thrombosis and Haemostasis of the Scientific and Standardization Committee (ISTH SCC) [15]. Arterial and venous thrombotic complications required positive imaging.

Two authors reviewed all the EMR data independently to minimize bias and discrepancies were resolved with discussion. The data were then processed and analyzed without personal identifiers to maintain patient confidentiality in adherence to the Health Insurance Portability and Accountability Act (HIPAA).

### Outcomes and statistical analysis

Patients were stratified into four groups based on their BMI at diagnosis: BMI 18.5–24.9 kg/m^2^ (normal weight), BMI 25–29.9 kg/m^2^ (overweight), BMI 30–39.9 kg/m^2^ (obesity class I and II), BMI more than 40 kg/m^2^ (obesity class III or severe obesity). BMI stratification was based on the Centers for Disease Control and Prevention (CDC) [16]. The primary endpoint was platelet count at diagnosis. The secondary endpoints included platelet nadir, treatment requirement, days to achieve response, steroids dependency, refractoriness, number of lines of therapy, response to initial treatment, duration of initial response (months), clinically relevant non-major (CRNM) bleeding, major bleeding and thrombosis.

The ANOVA test was used to compare the continuous variables, while Chi-square or Fisher’s tests were used for categorical variables as appropriate for number of cases involved. The overall response duration to initial treatment was calculated by Kaplan-Meier model. Statistical significance was assumed for a two-sided alpha of *≤* 0.05. To investigate the baseline variables related with platelet count at diagnosis, the ordinal logistic regression model was employed. Platelet count was treated as an ordinal variable, categorized by severity into three groups: 0–50 × 10^9^/L, 50–100 × 10^9^/L, 100–150^9^/L. The lowest category, 0–50 × 10^9^/L, served as the reference for comparative analysis against the other severity groups. Both univariate and multivariate analyses were conducted to explore the relationships between the variable of interest and ordinal platelet count. Logistic regression model was used to determine the baseline variables associated with treatment requirements, controlling for BMI when analyzing other associations. Logistic regression analysis results are presented as the odds ratio (OR) with a 95% confidence interval (CI). All analyses were performed using R program software (R version 4.1.3, Vienna).

## Results

### Baseline demographic information

**Fig. 1:**
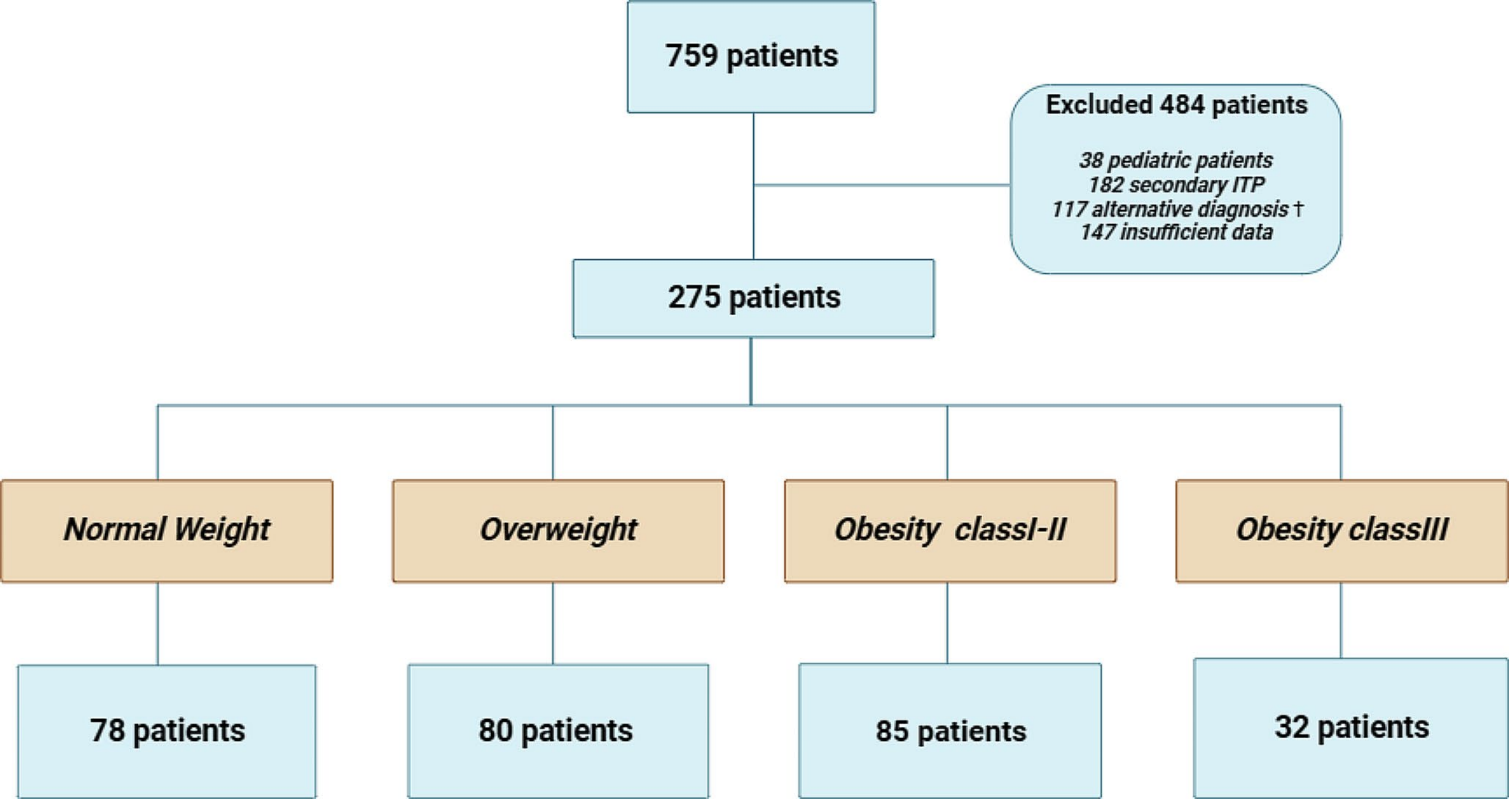
Alternative diagnoses including liver disease, bone marrow failure, myelodysplastic syndrome, acute myeloid leukemia, chemotherapy-induced thrombocytopenia, hypersplenism

Using the keywords of “thrombocytopenia”, “immune thrombocytopenic purpura” and “idiopathic thrombocytopenic purpura” 759 patients were identified. After reviewing the medical records, the final analysis consisted of 275 patients; median follow-up was 44 (2-144) months for the whole cohort and was 90 (1-144) months, 58 (1-139) months, 36 (1-144) months and 20 (1-120) for normal BMI weight, overweight, obesity classI-II, obesity class III respectively. Reasons for exclusion are detailed in Fig. 1, including 38 pediatric patients, 182 patients with secondary ITP, 117 diagnoses other than ITP, and 147 patients with insufficient or missing BMI data. The median age at diagnosis for the whole group was 50 years old with slight female predominance (*N* = 168, 61.1%). 78 patients, 80 patients, 85 patients and 32 patients were included in BMI 18.5–24.9 kg/m^2^, 25–29.9 kg/m^2^, 30–39.9 kg/m^2^ and > 40 kg/m^2^, respectively (Fig. 1). In addition, the EMR at Montefiore Medical Center revealed 631,760 patients having a BMI between 30 and 39.9 kg/m^2^, and 168,531 patients with a BMI exceeding 40 kg/m^2^ were identified. There was no difference in age at diagnosis and gender within the four groups. There was a higher proportion of black and Hispanic patients in the higher BMI group (*p* = 0.044). (Table 1)

**Table 1:** BMI-defined baseline characteristics, treatments and outcomes

BMI kg/m^2^	18.5–24.9	25-29.9	30-39.9	> 40	*P* value
	*N* = 78	*N* = 80	*N* = 85	*N* = 32	
Mean Age (SD)	52.1 (20.8)	53.8 (18.3)	52.4 (17.9)	43.1 (18.0)	0.054
Female, n (%)	40 (51.3%)	49 (61.3%)	57 (58.7%)	22 (68.7%)	0.156
Race, n (%)					**0.044**
White	36 (46.2%)	33 (41.2%)	23 (27.1%)	6 (18.8%)	
Black	9 (11.5%)	12 (15.0%)	15 (17.6%)	12 (37.5%)	
Hispanic	26 (33.3%)	26 (32.5%)	36 (42.2%)	12 (37.5%)	
Others	7 (9.0%)	9 (11.2%)	11 (12.9%)	2 (6.3%)	
Mean platelet level, x10^9^/L (SD)					
At diagnosis	80.8 (49.2)	61.5 (45.6)	52.7 (47.9)	28.7 (30.9)	**< 0.001**
Nadir	65.2 (44.8)	46.9 (45.0)	40.6 (52.1)	15.2 (19.1)	**< 0.001**
Treatment requirement, n (%)	27 (34.6%)	43 (53.8%)	59 (69.4%)	30 (93.8%)	**< 0.001**
Dexamethasone as initial therapy, n (%)	17 (63.0%)	25 (58.1%)	35 (59.3%)	21 (70.0%)	0.705
Days to achieve response, mean (SD)	4.3 (2.9)	5.0 (2.3)	5.2 (7.1)	6.2 (8.0)	0.690
Lines of treatment, n (SD)	1.7 (0.9)	2.5 (1.6)	2.7 (1.5)	2.7 (1.6)	**0.032**
Steroid dependent, n (%)	4 (14.8%)	9 (21.4%)	21 (35.0%)	13 (43.3%)	**0.048**
Refractoriness, n (%)	7 (25.9%)	19 (46.3%)	26 (44.1%)	15 (50.0%)	0.253
Response to initial therapy, n (%)					0.984
CR	18 (69.2%)	28 (66.7%)	33 (63.5%)	19 (70.4%)	
PR	6 (23.1%)	10 (23.8%)	12 (23.1%)	5 (18.5%)	
NR	2 (7.7%)	4 (9.5%)	7 (13.4%)	3 (11.1%)	
Response duration, median months	Not reached	11	2	5	**0.009**
CRNM/Major Bleeding, n (%)	5 (6.41%)	6 (7.5%)	9 (10.6%)	8 (25.0%)	0.422
Thrombosis, n (%)					0.620
Venous	0 (0.0%)	3 (7%)	5 (5.9%)	1 (3.1%)	
Arterial	1 (3.7%)	3 (7%)	0 (0.0%)	2 (6.2%)	

### Obesity is associated with lower platelet counts

Mean platelet counts at diagnosis, and at nadir negatively correlated with the patient’s BMI. At diagnosis, normal BMI had mean platelet counts of 80.8 × 10^9^/L, while the overweight, obesity class I-II, and obesity class III were significantly lower at 61.5, 52.7, 28.7 × 10^9^/L, respectively (*p* < 0.001). The same was true for the nadir counts throughout the course (normal weight: 65.2 × 10^9^/L; overweight: 46.9 × 10^9^/L; obesity class I-II: 40.6 × 10^9^/L; obesity class III: 15.2 × 10^9^/L, *p* < 0.001, Table 1).

### Obesity is associated with adverse ITP outcomes

**Fig. 2:**
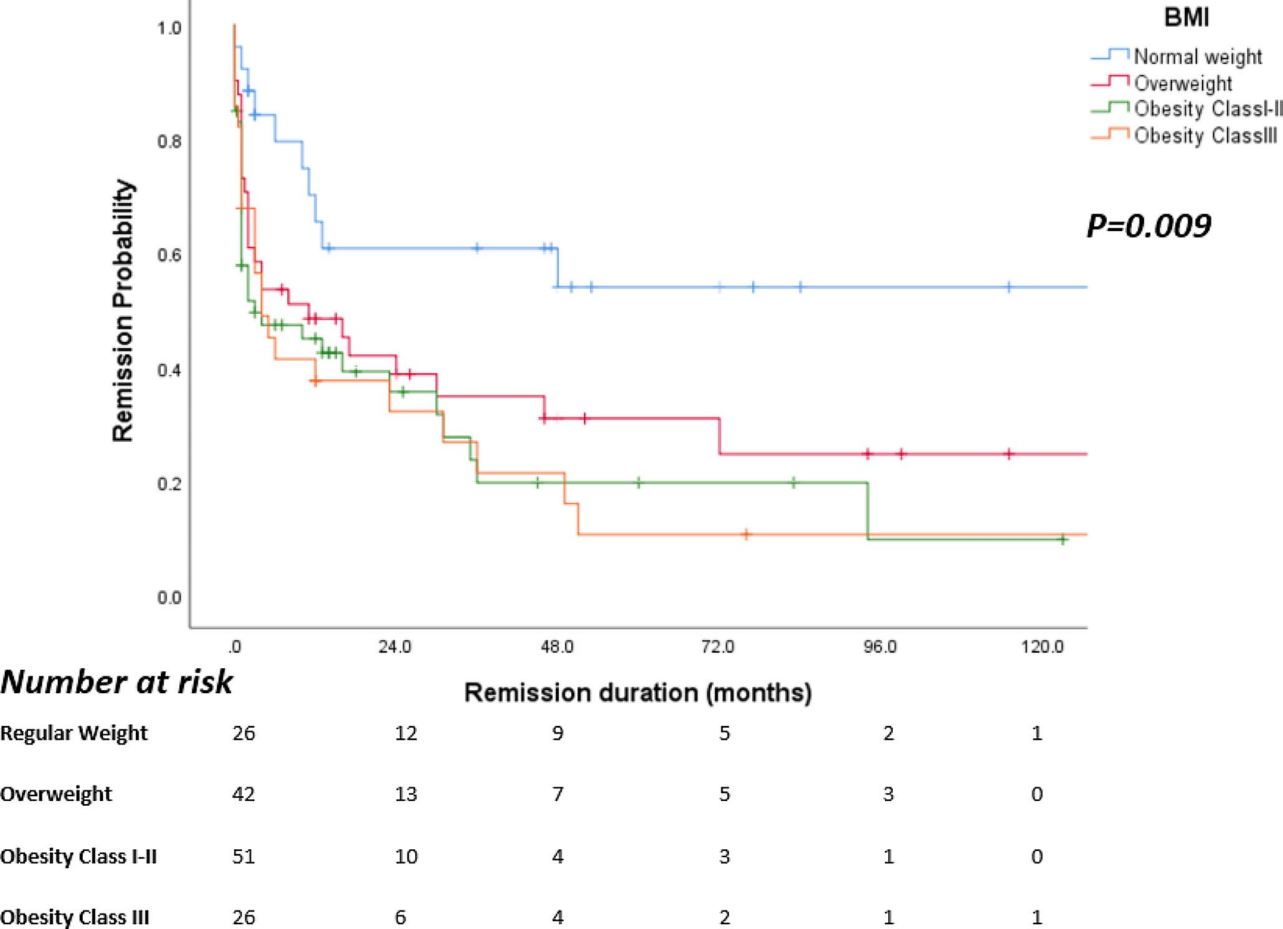
Remission duration for each group

Patients with higher BMI were more likely to require ITP-directed therapy to maintain platelet count in a safe range (normal weight: 34.6%, overweight: 53.8%, obesity class I-II: 69.4%, obesity class III: 93.8%, *p* < 0.001, Table 1). There was no difference between the type of initial corticosteroid therapy among the four groups. Patients with high BMI also required more lines of therapy to stabilize their platelet count (1.7, 2.5, 2.7, and 2.7, mean lines of treatment for normal weight, overweight, obesity class I-II, and obesity class III, respectively, *p* = 0.032) (Table 1). The incidence rate ratio was also calculated by Poisson regression, follow-up time was used as offset. Compared to the normal weight group, the incidence rate ratio for overweight, obesity class I-II and obesity class III were 3.98, 4.82 and 6.60, respectively. Further details regarding the treatments administered to each BMI group can be found in Table 2. Among the patients who received corticosteroids as first-line therapy, obese patients were more corticosteroid-dependent (*p* = 0.048) and had a shorter duration of remission compared to the non-obese patients (median response duration for normal weight vs. overweight, obesity class I-II, obesity class III respectively not reached vs. 11 months, 2 months, 5 months *p* = 0.009) (Fig. 2). There was no difference in depth of response, time to response, refractoriness, major and CRNM bleeding and thrombotic complications among the groups (Table 1).

**Table 2:** Subsequent therapies administered in each group

		BMI (kg/m^2^)			
		**18.5–24.9**	**25-29.9**	**30-39.9**	**> 40**
IVIG		10	22	30	16
Rituximab		3	14	23	14
Romiplostim		6	9	17	7
Eltrombopag		0	14	17	7
Avatrombopag		0	1	2	0
Splenectomy		1	9	15	6
Fostamatinib		0	1	2	1
Cyclosporin		0	2	0	0
Danazol		0	4	1	1
Mycophenolate mofetil		0	1	0	1
Azathioprine		0	0	1	0
Dapsone		0	1	0	0
Vincristine		0	1	1	0
WinRho		0	1	0	2

In univariate and multiple regression analyses, both BMI and female gender were significantly associated with lower platelet count at diagnosis. Univariate analysis also demonstrated an association of BMI and female gender with treatment requirement. However, in multivariate analysis, only BMI remained still significantly associated with need for therapy (Table 3).

**Table 3:** Logistic univariable and multivariable analysis for platelet level at diagnosis and treatment requirement (Reference: platelet count: 0–50 × 10^9^/L)

	Platelet Counts at Diagnosis				Treatment Requirement			
	Univariate analysis		Multivariate analysis		Univariate analysis		Multivariate analysis	
	OR (95% CI)	P	OR (95% CI)	P	OR (95% CI)	P	OR (95% CI)	P
**Age**	1.01 (0.99–1.02)	0.38			0.99 (0.97–1.01)	0.16		
**Female**	0.4 (0.22–0.73)	**< 0.01**	0.44 (0.24–0.82)	**0.01**	1.86 (1.13–3.04)	**0.01**	1.15 (0.96–2.76)	0.075
**Ethnicity**								
**White**	1				1			
**Black**	0.73 (0.32–1.68)	0.46			1.21 (0.60–2.42)	0.63		
**Hispanic**	0.5 (0.25-1.00)	0.05			1.67 (0.94–2.96)	0.07		
**Other**	0.62 (0.23–1.64)	0.33			1.53 (0.66–3.59)	0.37		
**BMI kg/m** ^**2**^								
**18.5–24.9**	1				1		1	
**25-29.9**	0.51 (0.24–1.09)	0.08			2.19 (1.15–4.17)	**0.02**	2.28 (0.71–7.35)	0.17
**30-39.9**	0.36 (0.17–0.77)	**0.01**	0.40 (0.19–0.88)	**0.02**	4.28 (2.22–8.26)	**< 0.01**	5.06 (1.55–16.59)	**0.01**
**>40**	0.030 (0-0.30)	**< 0.01**	0.04 (0.01–0.34)	**< 0.01**	28.33 (6.28-127.68)	**< 0.01**	22.02 (3.24-149.56)	**< 0.01**

## Discussion

This is the largest study to evaluate association between obesity and adverse ITP outcomes. The overall prevalence of ITP is estimated at 9.5 per 100,000 (0.0095%) adults in the US [17]; our study, with stringent exclusion criteria and excluding secondary ITP cases revealed a prevalence of 85 per 631,760 (0.013%) for obesity class I-II and 32 per 168,531 (0.019%) for obesity class III, surpassing previously reported figures. Our higher prevalence may be secondary to the increased incidence of more severe thrombocytopenia in our obese Bronx population leading to an ability for earlier and better detection. Furthermore, while not reaching statistical significance, individuals with severe obesity exhibited an earlier onset of the disease compared to those with lower BMI values. Bleeding was mostly minor, with the number of CRNMB and major bleeding events not different between groups. Bleeding is seen more frequently in older patients with ITP [18], and our patient population was younger with a median age of 50, similar to mean age of 50 to 55 years old at ITP diagnosis reported in the literature [1, 2].

Though ITP patients have a high initial response rate to corticosteroids, eventual treatment failure or corticosteroid dependency can be as high as 80% [19]. Given the high treatment failure rate, it is important to identify risk factors for relapse and predictors of response to therapy. Previously, an elevated ferritin level, positivity for Hepatitis B Surface Antigen (HBsAg) or for antinuclear antibody (ANA), platelet counts ≥ 20 × 10^9^/L and older age were identified as adverse factors for adult ITP relapse and response to treatment outcomes [18, 20, 21], while age > 6 yrs, higher platelet count at diagnosis and lower absolute lymphocyte count were identified as adverse risk factors in pediatric ITP outcomes [22, 23]. Results of our study suggest that obesity may also be a risk factor for diagnosis and relapse in adults.

In our study, we observed that obese patients who responded to therapy had a shorter remission duration than non-obese patients and more likely to require more lines of treatment. We also demonstrated that given the lower platelet counts, our patients were more likely to require therapy. ITP guidelines recommend treatment when the platelet is lower than 20–30 × 10^9^ /L [24]. In our study, we found that obese patients had significantly lower platelet counts, which understandably can lead to a higher likelihood of treatment requirement. In a comparable study carried out in China, there was no evidence indicating that obesity had an impact on platelet count [9]. One potential explanation for this disparity is that our study cohort includes patients with higher BMIs in general and different racial backgrounds, with the majority of our patient population represented by Hispanic and Black patients.

Several mechanisms and hypotheses might explain the lower platelet counts in obese patients. Obesity is a chronic inflammatory disorder, associated with adipocyte hypertrophy and secretion of pro-inflammatory cytokines, such as interleukin (IL)-6, IL-1, IL-8, and tumor necrosis factor (TNF)-α, as well as adipokines such as leptin, adipsin, resistin, and visfatin by adipose tissue [25, 26]. Increased production of IL-6 promotes hepatic synthesis and secretion of C-reactive protein (CRP). CRP levels have been shown to be elevated in thrombocytopenic patients, inversely correlating with platelet counts [27]. In a recent in vitro study, CRP has been shown to bind to the Fc receptor (FcR), amplifying the antibody-mediated phagocytosis and enhancing cellular destruction [28]. Leptin is an adipokine that acts via transmembrane receptors which are structurally similar to the class I family of cytokine receptors, including receptors for IL. Leptin promotes the secretion of multiple inflammatory cytokines and in turn, inflammatory cytokines can increase leptin expression to create a chronic inflammatory environment via positive feedback mechanism [29]. Leptin levels were found to be higher in ITP patients than in healthy controls, and inversely related to platelet counts. Furthermore, a positive association was observed between leptin levels and the platelet associated IgG level [30] and elevated leptin levels were demonstrated to stimulate the secretion of IgG antiplatelet antibodies by splenocytes and peripheral blood mononuclear cells from patients with chronic ITP. Furthermore, leptin may also promote the generation of platelet-reactive T cells [31]. Adipsin, secreted by adipocytes, is a serine protease with similar activity to complement factor D. It can cleave complement factor B, thereby activating the alternative complement pathway [32]. Complement activation may contribute to platelet destruction in ITP, even when autoantibodies are not detectable [33] and complement activation is associated with lower platelet counts and immature platelet fraction in individuals with ITP [34]. Recently, complement inhibition has been demonstrated to have therapeutic efficacy in ITP [35]. Lastly, IL -17/IL-10 ratios are skewed in heavier patients and may make them more resistant to steroid therapy [36–38].

In our findings, we also observed that obese patients are more corticosteroids dependent and experience shorter remission durations following corticosteroid therapy. Choice of steroid therapy for different BMIs is unlikely to explain our findings since we did not find a difference in the choice of steroids administered among the groups(*p* = 0.705). Inadequate steroid dosing is also unlikely: dosing was weight-based in all beside two patients in the obesity class I-II group and three patients in the obesity class III group. These 5 patients received a fixed dose of 60 mg of prednisone instead of the recommended 1 mg/kg. In contrast to other studies, we did not find that age is a risk factor for ITP [18, 20]. It’s possible that the reason for this is due to the relatively younger age of our patients compared to the average age of patients with ITP.

Autoimmune disorders are more prevalent among the women [39] and ITP is well known to affect more women than men [2, 40–42]. Our study supports these data and also demonstrate that female gender affects ITP outcomes negatively. The proposed causative hypotheses include sex hormones, higher levels of antibody production, more circulating T cells, and the overexpression of vestigial-like family member 3 (VGLL3), a transcription cofactor that leads to the activation of pro-inflammatory genes, including B-cell activating factor, and can promote enhanced B-cell activation in women [43, 44]. However, how gender affects ITP outcomes remains controversial.

The Bronx has the highest percentage of obese adults of all NYC boroughs with obesity being more common among women and individuals of lower socioeconomic status [45]. The economic burden from ITP is extremely high especially during the first 12 months of diagnosis [13]. Patients reduce their work hours and productivity due to ITP diagnosis and symptoms [12]. The inferior outcomes of ITP in obese patients could be a vicious cycle in this particular population and contribute to this increased vulnerability.

The advantages of our study are its large size and its diversity. We meticulously ensured the inclusion of individuals from all our patients, including those with diverse ethnic, demographic backgrounds. This is the largest study to demonstrate the relationship between obesity and clinical and treatment ITP outcomes, and to examine these aspects for diverse, underserved, and underrepresented populations. Despite the inherent potential for bias in a retrospective study, we took steps to mitigate this by using direct patient electronic medical record review with two separate data researchers and using objective outcome criteria, rather than assessing “big data”, we were able to perform in-depth analysis of therapies and adverse outcomes and ensure accurate diagnoses, therapies and responses. We hope that our findings of a strong association of obesity with ITP diagnosis and response to therapy will pave the way for future prospective clinical trials exploring weight loss modalities as an adjunct therapy for ITP treatment.

## Data Availability

No datasets were generated or analysed during the current study.
